# New insights into the health effects of dietary saturated and omega-6 and omega-3 polyunsaturated fatty acids

**DOI:** 10.1186/1741-7015-10-50

**Published:** 2012-05-21

**Authors:** Michel de Lorgeril, Patricia Salen

**Affiliations:** 1 Laboratoire Cœur & Nutrition, TIMC-IMAG, Université Joseph Fourier-CNRS, Faculté de Médecine, Grenoble, France

## Abstract

Cardiovascular diseases and cancers are leading causes of morbidity and mortality. Reducing dietary saturated fat and replacing it with polyunsaturated fat is still the main dietary strategy to prevent cardiovascular diseases, although major flaws have been reported in the analyses supporting this approach. Recent studies introducing the concept of myocardial preconditioning have opened new avenues to understand the complex interplay between the various lipids and the risk of cardiovascular diseases. The optimal dietary fat profile includes a low intake of both saturated and omega-6 fatty acids and a moderate intake of omega-3 fatty acids. This profile is quite similar to the Mediterranean diet. On the other hand, recent studies have found a positive association between omega-6 and breast cancer risk. In contrast, omega-3 fatty acids do have anticancer properties. It has been shown that certain (Mediterranean) polyphenols significantly increase the endogenous synthesis of omega-3 whereas high intake of omega-6 decreases it. Finally, epidemiological studies suggest that a high omega-3 to omega-6 ratio may be the optimal strategy to decrease breast cancer risk. Thus, the present high intake of omega-6 in many countries is definitely not the optimal strategy to prevent cardiovascular disease and cancers. A moderate intake of plant and marine omega-3 in the context of the traditional Mediterranean diet (low in saturated and omega-6 fatty acids but high in plant monounsaturated fat) appears to be the best approach to reduce the risk of both cardiovascular diseases and cancers, in particular breast cancer.

## Introduction

Cardiovascular disease (CVD) is a leading cause of death in most countries. Reducing saturated fatty acid (SFA) intakes is still at the heart of dietary recommendations to reduce CVD, mainly because of its effect on blood cholesterol [1]. This view has recently been challenged. First, a review of epidemiological studies failed to conclude that SFAs are associated with an increased risk of CVD [2]. Second, the validity of meta-analyses of clinical trials showing that CVD can be prevented by replacing SFAs with polyunsaturated fatty acids (PUFAs) has been questioned [3,4] because they omitted relevant trials with unfavorable outcomes (selection bias) and included others that were poorly designed (no randomization) [5,6]. Third, it has been claimed that the effect of diet on a single biomarker (such as plasma cholesterol) is insufficient evidence to assess CVD risk [7]. Fourth, the hypothetical protective effect of omega-6 PUFAs has been said to be considerably exaggerated [8,9], because of the failure to draw a line between the trials that selectively increased omega-6 PUFAs and those that substantially increased omega-3 PUFAs - known to reduce CVD risk [10,11] - together with omega-6 PUFAs to replace SFAs [3,4]. Finally, clinical and epidemiological studies exploring the dietary fat issue failed to provide a clear biological understanding of the effect of the various dietary fats on the risk of CVD.

There is one exception: the Mediterranean diet [12], which is a complex interplay between the different series of dietary lipids, including conjugated or non-conjugated (animal or industrial) trans fatty acids, short-, medium- and long-chain SFAs, various series - at least the omega-7 and omega-9 - of monounsaturated fatty acids, and the various series of PUFAs, including omega-3 and omega-6 [12,13]. All these lipids and their interactions should be taken into account when analyzing the effect of dietary fat on CVD complications and mortality. Besides the Mediterranean diet, this complexity makes the interpretation of epidemiological data very difficult and explains the ceaseless controversy about dietary fats and the risk of CVD. However, recent studies in experimental nutrition using the concept of myocardial preconditioning [14] have provided new and critical insights into the biological effects of dietary fat on CVD complications and mortality.

## Dietary fat, myocardial preconditioning and cardiovascular disease

Preconditioning - that is, the ability of the myocardium to withstand an ischemia-reperfusion injury - is a major concept in cardiology [14]. The extent of cell death during and after myocardial ischemia is actually the primary determinant of the outcome of a heart attack. Ventricular arrhythmia and cardiac pump failure are among the main clinical complications that are prevented by the initiation of myocardial preconditioning. Drug makers have failed to identify pharmacological methods able to induce chronic preconditioning [15]. In contrast, there are strong clinical and experimental data suggesting that lifestyle - including moderate alcohol drinking and physical exercise - is a potent preconditioner [16,17]. Also, polyphenols present in certain plants - and abundant in red wine - induce preconditioning [18]. Although obtained in experimental conditions, data regarding chronic myocardial preconditioning as induced by lifestyle and nutrition are highly consistent with our general clinical knowledge regarding the effects of lifestyle and nutrition on CVD complications and mortality. The next question is whether myocardial preconditioning could shed light on the role of dietary fat in CVD.

In fact, two recent studies on rat models have provided major findings by comparing the effects of different dietary fat profiles on the induction of myocardial preconditioning [19,20]. In both studies, the investigators compared the effects of diets that were high in SFAs or omega-6 but poor in omega-3 with diets that were poor in SFAs and omega-6 but rich in omega-3. In both studies, the best protection was obtained in the groups of rats receiving the diet high in omega-3 PUFAs but relatively poor in SFAs and omega-6 PUFAs, while the diet rich in omega-6 but relatively poor in SFAs and omega-3 PUFAs provided no protection [19] or a protection halfway between the diets rich in SFAs, on one hand, and in omega-3, on the other hand [20]. Thus, as compared with the common Western diet - rich in either SFAs or omega-6 PUFAs but poor in omega-3 PUFAs - an optimal dietary pattern aimed at reducing CVD complications and mortality should include a reduced intake of both SFAs and omega-6, in addition to increased plant and marine omega-3 PUFAs. Not surprisingly, this dietary fatty acid profile is similar - but not identical - to that of the Mediterranean diet, which also is rich in plant monounsaturated fat and poor in industrial trans fatty acids [12,13].

These data should help identify the optimal dietary fatty acid profile to reduce the risk and the complications of CVD. Thus, maintaining high [8] or increasing - as proposed by certain experts [6,9] - the intake of omega-6 in lieu of SFAs is definitely not the optimal strategy to prevent CVD complications.

## Dietary fat and cancer

In animal studies, omega-6 PUFAs have a strong mammary tumor-enhancing effect [21,22]. In order to exert their carcinogenic effects, they must first undergo an oxidative metabolization, mainly through the lipoxygenase and cyclooxygenase pathways [23,24]. The main substrate of these oxidative pathways is arachidonic acid, which is mainly produced from dietary linoleic acid, the most common omega-6 PUFAs in Western foods and cooking fats. Several recent epidemiologic studies have found a positive association between dietary omega-6 PUFAs and breast cancer risk [25-30]. Certain analyses took into account a genetic predisposition related to omega-6 metabolism. To determine whether 5-lipoxygenase (LOX)-mediated dietary omega-6 metabolism might influence breast cancer risk, investigators examined genetic variants of the LOX enzyme in combination with linoleic acid intakes [25]. They found that women with a genetic aberration affecting the LOX enzyme whose diet provided a high level of omega-6 (linoleic acid) had a significantly increased breast cancer risk [25]. However, when women with the same high-risk genetic profile had a diet lower in linoleic acid, their genotype had no significant effect on their breast cancer risk. This demonstration that a diet-gene interaction increases the risk of cancer may explain why some previous studies were inconsistent or conflicting. Other recent studies have shown interactions between heterocyclic amines and omega-6 PUFAs on the one hand [26], and between omega-3 and omega-6 on the other hand [27], in determining the risk of invasive breast cancer. Other factors, such as the obesity status [28], were shown to affect the association between dietary PUFAs and breast cancer risk. Finally, the food sources of omega-3 and omega-6 PUFAs, as well as their relative amounts in the diet of individuals, appear to be very important for breast cancer risk [29,30].

Thus, there are several recent and concordant studies that strongly suggest that dietary omega-6 PUFAs - the consumption of which is encouraged worldwide to decrease blood cholesterol - increase the risk of breast cancers. In the same line of reasoning, it is important to recall that the most frequently prescribed cholesterol-lowering drugs (including statins) increase the blood concentration of arachidonic acid, the main omega-6 PUFA in cell membranes [31]. Also, studies have suggested that low cholesterol and/or cholesterol-lowering are associated with an increased risk of cancers [32]. Thus, despite the fact that many confounders tend to obscure the effects of cholesterol-lowering drugs on the clinical occurrence of cancers, the association of high intake of omega-6 and statins - both aimed at reducing blood cholesterol to prevent CVD - may add up to increase cancer risk, in particular breast cancer risk. Further studies are urgently needed to explore the issue.

It could be said that most data regarding the effects of dietary omega-6 PUFAs on cancers are observational [25-30] and do not demonstrate cause-effect relationship. Only randomized trials can demonstrate causality. In fact, two dietary trials not initially designed to test a diet-cancer hypothesis, the Veterans Los Angeles trial [33] and the Lyon Diet Heart Study [34], provided some information about dietary omega-6 and cancers. In the Los Angeles trial, there was a huge increase in dietary omega-6 in the experimental group compared with the control group (15% total energy versus 5%) and there was a significant increase in the incidence of new cancers and in cancer mortality in the high omega-6 group [33]. By contrast, in the Lyon trial, the intake of omega-6 was slightly but significantly reduced in the experimental group (3.6% total energy versus 5.3%) and there was a significant decrease in cancer incidence in the low omega-6 group [34]. These two trials with very different amounts of omega-6 in the experimental groups do not definitely demonstrate that omega-6 fatty acids per se increase the risk of cancer - other dietary factors and various interactions (including drug treatment as mentioned above) likely played a role in the clinical surfacing of new cancers and their severity - but they clearly signal that lipid profiles with high or relatively high (> 5% energy) omega-6 tend to increase the risk of cancers, which is in line with the observational studies discussed above.

At the same time, omega-3 PUFAs were shown to have chemopreventive properties against various cancers and their complications, including colon and breast cancer [35,36]. It is therefore important to design dietary strategies that will result in increased omega-3 in diet, blood and tissues, associated with decreased omega-6. In addition to increasing the dietary intake of omega-3, it is possible to stimulate the endogenous synthesis of very long-chain omega-3 PUFAS - often called 'marine' omega-3 - out of their plant substrate alpha-linolenic acid, through the consumption of plant pigments such as the polyphenols found, for instance, in grapes and red wine [37-39]. Both alpha-linolenic acid and the polyphenol anthocyanins are present in quite large amounts in the traditional Mediterranean diet, also poor in omega-6, which may at least partly explain the remarkable protection this diet provides against cancers [40,41]. In contrast, the main dietary omega-6 linoleic acid inhibits synthesis and cell incorporation of long-chain omega-3 PUFAs [42,43] which is in line with the effects of omega-6 on cancer risk as discussed above.

The association of oleic acid - the main fatty acid of olive oil, a major component of the Mediterranean diet - with breast cancer risk has been analyzed in several studies [44-46] and provided conflicting data. In fact, it is only when the level of oleic acid in blood or cells was used in the analyses (and not as a nutrient through a frequency questionnaire) that it was positively associated with cancer risk [46]. The level of oleic acid in blood and tissue is more dependent on endogenous metabolism than on dietary intake. The main enzymatic system regulating oleic acid level is the delta-9 desaturase - also called stearoyl-coenzyme A desaturase - and its activity depends on dietary (carbohydrate intake), hormonal (insulin) and lifestyle (physical exercise) factors [47]. Thus, blood concentration of oleic acid is not a surrogate of the consumption of oleic acid but rather a biomarker of lifestyle associated with insulin resistance, which is by itself positively associated with the risk of breast cancer [48]. Finally, dietary oleic acid is not necessarily a marker of olive oil consumption as it is also one of the main fatty acids of meat. It is critical in epidemiologic studies analyzing the relationships between oleic acid intake and any clinical outcome to include the geographic area of the studied population: in the Mediterranean area, the dietary source of oleic acid is mainly olive oil (a plant food) whereas in most Western countries, animal foods are the main sources of oleic acid. Importantly, olive oils contain more than the sole lipids; certain phytonutrients such as polyphenols may also interfere with the risk of cancers [49]. Thus, when analyzing the relationships between breast cancer and dietary habits, the type of fatty foods - plant versus animal - is as important as the type of fatty acids.

## Summary and prospects

From the most recent experimental and epidemiological studies, we conclude that the optimal dietary fat pattern to reduce the risk of both CVD and most cancers should include a low intake of SFAs and omega-6 PUFAs. Small amounts (1% to 2% of energy intake) of the essential linoleic acid - easy to find in most Western foods - are sufficient to prevent omega-6 deficiency [50,51]. The amounts of omega-6 in most Western foods are so high that it could be difficult to obtain an intake of omega-6 lower than 4% of energy [52], which would probably be the optimal level. The high average intake of omega-6 PUFAs in Western countries [53,54] may explain the persistently high rate of CVD complications and the increased incidence of certain cancers, including breast cancer. The intake of omega-3 PUFAs, from plant and marine sources, should be moderate (a minimum of 3 g/day in average for an adult with at least 2 g/day of the essential alpha-linolenic acid), which is far from the case at present in many populations [53,54].

Finally, regarding the intake of oleic acid, it is critical to differentiate the food sources, since the health effects of oleic acid obtained from meat or from olive oil are different. To simplify the dietary advice aimed at protecting health - and help consumers to understand it - the best approach is probably the traditional Mediterranean diet model. No dietary pattern has been so extensively studied, and no other has been shown to provide so many benefits without any adverse effects.

## Abbreviations

CVD: cardiovascular disease; LOX: 5-lipoxygenase; PUFA: polyunsaturated fatty acid; SFA: saturated fatty acid.

## Competing interests

The authors declare that they have no competing interests.

## Authors' contributions

MdeL drafted the manuscript. PS critically revised the manuscript and gave final approval for publication.

## Pre-publication history

The pre-publication history for this paper can be accessed here:

http://www.biomedcentral.com/1741-7015/10/50/prepub
